# Mutation Rate Variability across Human Y-Chromosome Haplogroups

**DOI:** 10.1093/molbev/msaa268

**Published:** 2020-10-13

**Authors:** Qiliang Ding, Ya Hu, Amnon Koren, Andrew G Clark

**Affiliations:** 1 Department of Molecular Biology and Genetics, Cornell University, Ithaca, NY; 2 New York Genome Center, New York, NY; 3 Department of Computational Biology, Cornell University, Ithaca, NY

**Keywords:** Y chromosome, haplogroup, mutation rate, DNA replication timing

## Abstract

A common assumption in dating patrilineal events using Y-chromosome sequencing data is that the Y-chromosome mutation rate is invariant across haplogroups. Previous studies revealed interhaplogroup heterogeneity in phylogenetic branch length. Whether this heterogeneity is caused by interhaplogroup mutation rate variation or nongenetic confounders remains unknown. Here, we analyzed whole-genome sequences from cultured cells derived from >1,700 males. We confirmed the presence of branch length heterogeneity. We demonstrate that sex-chromosome mutations that appear within cell lines, which likely occurred somatically or in vitro (and are thus not influenced by nongenetic confounders) are informative for germline mutational processes. Using within-cell-line mutations, we computed a relative Y-chromosome somatic mutation rate, and uncovered substantial variation (up to 83.3%) in this proxy for germline mutation rate among haplogroups. This rate positively correlates with phylogenetic branch length, indicating that interhaplogroup mutation rate variation is a likely cause of branch length heterogeneity.

The human Y chromosome provides unique opportunities for analyses in evolutionary genetics. Because the majority of its sequence does not undergo recombination, binary genetic markers, including single nucleotide variants, have been used to reconstruct the Y genealogy and assign males to haplogroups (Jobling and Tyler-Smith 2003). Haplogroup frequencies vary greatly across populations and have been used to trace patrilineal evolutionary history (Poznik et al. 2016).

An increasing number of studies sequenced the Y chromosome in cohorts (Wei et al. 2013; Yan et al. 2014; Hallast et al. 2015; Wei et al. 2018). Sequencing-based studies enable dating of divergence among Y-chromosome lineages. Such studies rely on the assumption that mutation rate among Y lineages is homogeneous. Mutation rate has been commonly estimated using deep pedigrees (Jobling and Tyler-Smith 2017). One study used a haplogroup O2 pedigree (Xue et al. 2009), whereas another study used 274 pedigrees (mostly haplogroups I and R) (Helgason et al. 2015). These estimates have been widely used, even when the subjects did not belong to the same haplogroup(s) in which the rate was estimated, under the assumption of that mutation rates are homogeneous among haplogroups (Kutanan et al. 2019).

The assumption of invariant mutation rate across haplogroups has been challenged by branch-length heterogeneity in Y-chromosome phylogenetic trees. In this phylogeny, different Y haplogroups showed significant differences in branch lengths (Yan et al. 2014; Hallast et al. 2015). For example, the branch lengths of haplogroup R samples are among the shortest. Because Y haplogroups display strong population substructure, previous studies favored explanations in which such heterogeneity resulted from nongenetic factors (e.g., paternal age variation). It was suggested that variation in Y-chromosome mutation rate across haplogroups was unlikely (Jobling and Tyler-Smith 2017).

In this study, we analyzed whole-genome sequences of >1,700 males in which DNA was from lymphoblastoid cell lines (LCLs). We defined a relative Y-chromosome somatic mutation rate, which was unlikely to be affected by nongenetic confounders. This rate showed significant variation across Y-chromosome haplogroups and exhibited positive correlation with phylogenetic branch lengths, supporting intrinsic interhaplogroup variation in Y-chromosome mutation rate. The patterns were reproduced in two independent data sets, strengthening their robustness. We suggest that Y-haplogroup-specific mutation rate estimates should be used when inferring divergence times from Y sequence.

## Results and Discussion

### Phylogenetic Branch Lengths Are Variable among Y-Chromosome Haplogroups

**Fig. 1.**
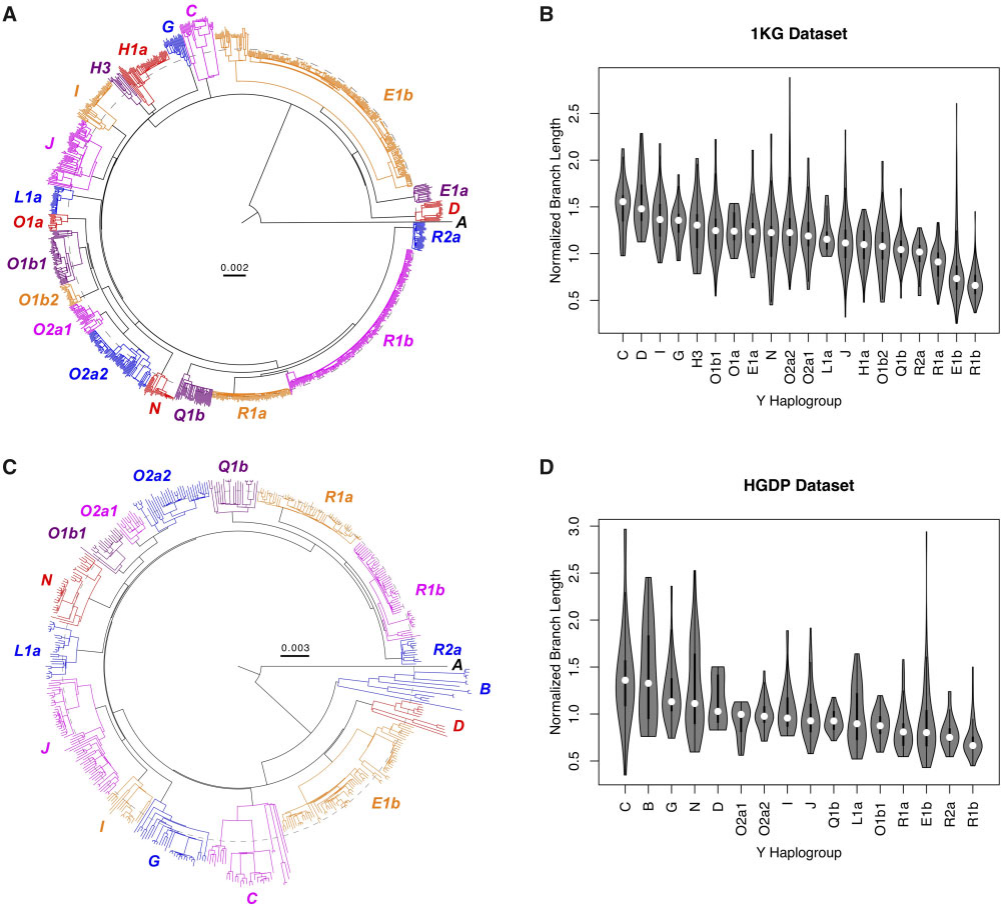
Y-chromosome phylogenetic branch length heterogeneity. (*A* and *C*) Y-chromosome phylogeny reconstructed using the 1KG (*A*) and HGDP (*C*) data sets. The phylogenies were reconstructed using the maximum-likelihood method in MEGA and visualized with FigTree. Haplogroups with more than ten samples were included, along with a sample from haplogroup A. Although all samples were collected at the present time, their branch lengths vary substantially, for example, haplogroup R1b samples have much shorter branch lengths (dashed circle) than samples from most other haplogroups. (*B* and *D*) Distribution of branch length (branch-specific evolution rate) by haplogroup in the 1KG (*B*) and HGDP (*D*) data sets. Evolutionary rates were estimated using the RelTime method and normalized to a mean of 1. Outlier samples (three in 1KG and two in HGDP) were removed. Substantial interhaplogroup evolutionary rate variation was observed, which was consistent between data sets (*ρ* = 0.81) and with (*A*) and (*C*).

To explore the reported pattern of branch length heterogeneity (Hallast et al. 2015), we analyzed the high-coverage Y-chromosome sequencing data of the 1000 Genomes Project (“the 1KG data set”). This data set contains 1,195 males from global populations (1000 Genomes Project Consortium 2015; Poznik et al. 2016), and has sufficient representation (more than ten samples) of 20 Y-chromosome haplogroups. We reconstructed a Y-chromosome phylogeny (fig. 1*A*), and rejected the hypothesis of evolutionary rate homogeneity throughout the tree (Kumar et al. 2012, 2018; Stecher et al. 2020). We subsampled data with one sample per haplogroup and uncovered significant interhaplogroup heterogeneity in evolutionary rate (likelihood-ratio test, *P* = 7.91 × 10^−41^), consistent with branch length variation (fig. 1*A*). These discoveries were robust to subsampling of individuals and nucleotide sites (supplementary note 1, Supplementary Material online). As a surrogate for branch length, we calculated normalized branch-specific evolutionary rate of terminal branches in the phylogeny using the RelTime method (Tamura et al. 2012). This method showed that the evolutionary rate varied greatly among haplogroups (fig. 1*B*), and the pattern of variation was consistent with figure 1*A* and previous reports (Hallast et al. 2015) (e.g., haplogroup R1b had shorter branch lengths than average).

To critically assess these findings, we analyzed the Y-chromosome sequencing data from the Human Genome Diversity Project (“the HGDP data set”) (Bergström et al. 2019). This data set contains 554 male samples and has sufficient representation of 16 Y-chromosome haplogroups. A phylogeny was reconstructed (fig. 1*C*), and the hypothesis of evolutionary rate homogeneity was rejected, even when using one sample per haplogroup (*P *= 3.93 × 10^−148^) and regardless of the subsampling (supplementary note 1, Supplementary Material online). We calculated branch-specific evolutionary rate as described above. Reassuringly, the pattern of variation observed in the HGDP data set (fig. 1*D*) was very similar to that of the 1KG data set. Taken together, we found that interhaplogroup branch length heterogeneity in human Y-chromosome trees does exist, whereas its cause requires further investigation.

### Within-Cell-Line Y-Chromosome Mutations Are Informative for Germline Mutational Processes

Previous studies suggested that branch length heterogeneity might be caused by nongenetic factors, for example, paternal age variation across populations, acting over many generations (Jobling and Tyler-Smith 2017). Another possibility is variation in mutation rate among Y-chromosome haplogroups. We propose to use “within-cell-line mutations” on the Y chromosome to distinguish the two possible causes. Within-cell-line mutations denote sites polymorphic in a given cell line found in the nonpseudoautosomal regions of the sex chromosomes in males, which indicates the presence of an alternative allele in a subset of cells. These mutations can only occur somatically along the hematopoietic lineage or in vitro during culturing of LCLs, and therefore are unlikely to be affected by nongenetic (e.g., cultural, demographic, and environmental) factors, including paternal age.

We identified within-cell-line mutations in the 1KG and HGDP data sets, requiring at least three reads supporting the alternative allele. We applied filters to remove false positives (supplementary Materials and Methods, Supplementary Material online). We resequenced the Y chromosome of five 1KG samples to ∼8-fold coverage, and successfully validated 92.9% (13/14) of within-cell-line mutations for which the alternative alleles were supported by five or more reads in the original samples. These mutations were selected for validation because the fraction of cells carrying the alternative alleles was likely higher, and thus more likely to also be present in the separately obtained samples used for resequencing.

**Fig. 2.**
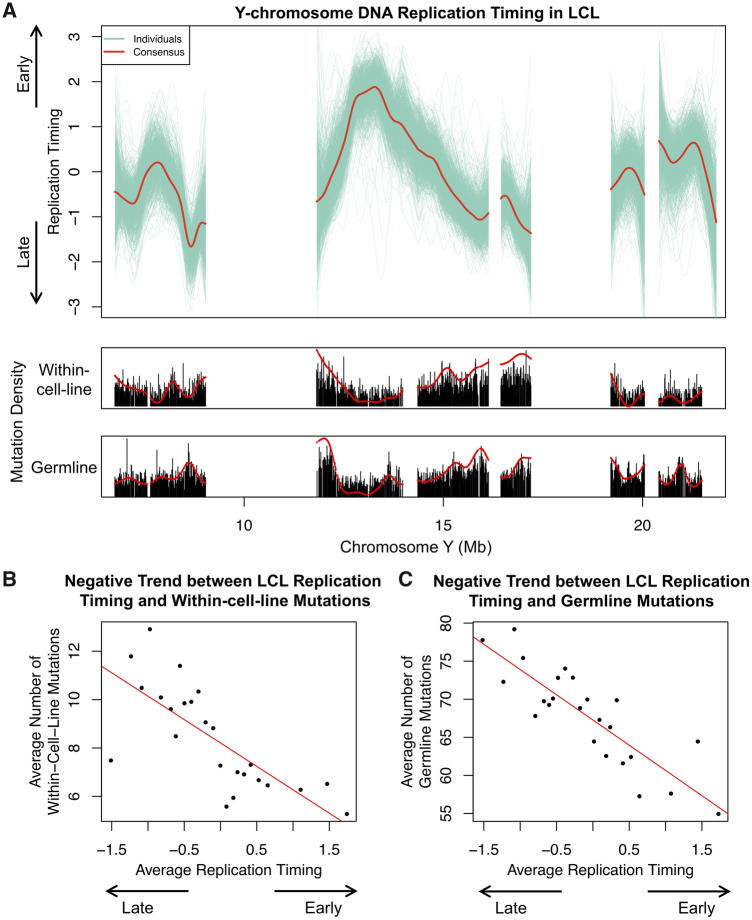
Y-chromosome replication timing and its relationship with mutations. (*A*) Top panel: replication timing profile of 1KG samples (green). Red: consensus. Bottom panel: mutation density tracks. Red: smoothed. (*B* and *C*) Y-chromosome replication timing shows a negative relationship with within-cell-line (*B*) and germline mutations (*C*). Replication timing windows were assigned to one of 25 bins based on timing. Mutation counts and timing of windows within each bin were averaged and plotted. Red, linear trend line.

DNA replication timing is correlated with the spatial distribution of mutations in humans (Stamatoyannopoulos et al. 2009; Koren et al. 2012), suggesting that replication timing can be used as a proxy for mutational processes. However, replication timing of the human Y chromosome has not been previously explored. We inferred a consensus Y-chromosome replication profile in LCLs using a method we described previously (Koren et al. 2014; Ding et al. 2020) (fig. 2*A*). Briefly, we inferred Y-chromosome replication timing for each cell line based on fluctuation of read depth across the chromosome. We averaged replication timing profiles for all samples to generate the consensus (supplementary Materials and Methods, Supplementary Material online). We uncovered a negative relationship between replication timing and within-cell-line mutation density (fig. 2*A* [lower panel] and *B*). Note that rigorously establishing statistical significance of this trend entails compensation for two sequential data sets that have distinctly different patterns of autocorrelation along the genome sequence. It is probably more convincing to note that the negative trend is robust to different approaches (supplementary note 2, Supplementary Material online).

Germline mutations (using SNPs in the 1KG data set) also showed negative relationship with LCL replication timing (fig. 2*A* [lower panel] and *C*). Germline and within-cell-line mutations have similar mutational spectrum (supplementary note 2, Supplementary Material online). These observations indicate that although mutational processes are known to differ between somatic cells (LCLs) and the germline, mutations in LCLs could nevertheless provide valuable insights into germline mutational processes.

### Substantial Variation in Y-Chromosome Somatic Mutation Rate across Haplogroups

As reasoned above, within-cell-line Y-chromosome mutations can be a valuable tool in distinguishing hypotheses regarding branch length heterogeneity. For a given sample, we define “relative Y-chromosome somatic mutation rate” as the counts of within-cell-line mutations on the Y chromosome (calculated as genetic distance using the Kimura two-parameter model) divided by that of the X chromosome. X-chromosome within-cell-line mutations were utilized to correct for non-Y-chromosome-specific factors affecting mutation accumulation, for example, variation in donor age and cell culturing.

**Fig. 3.**
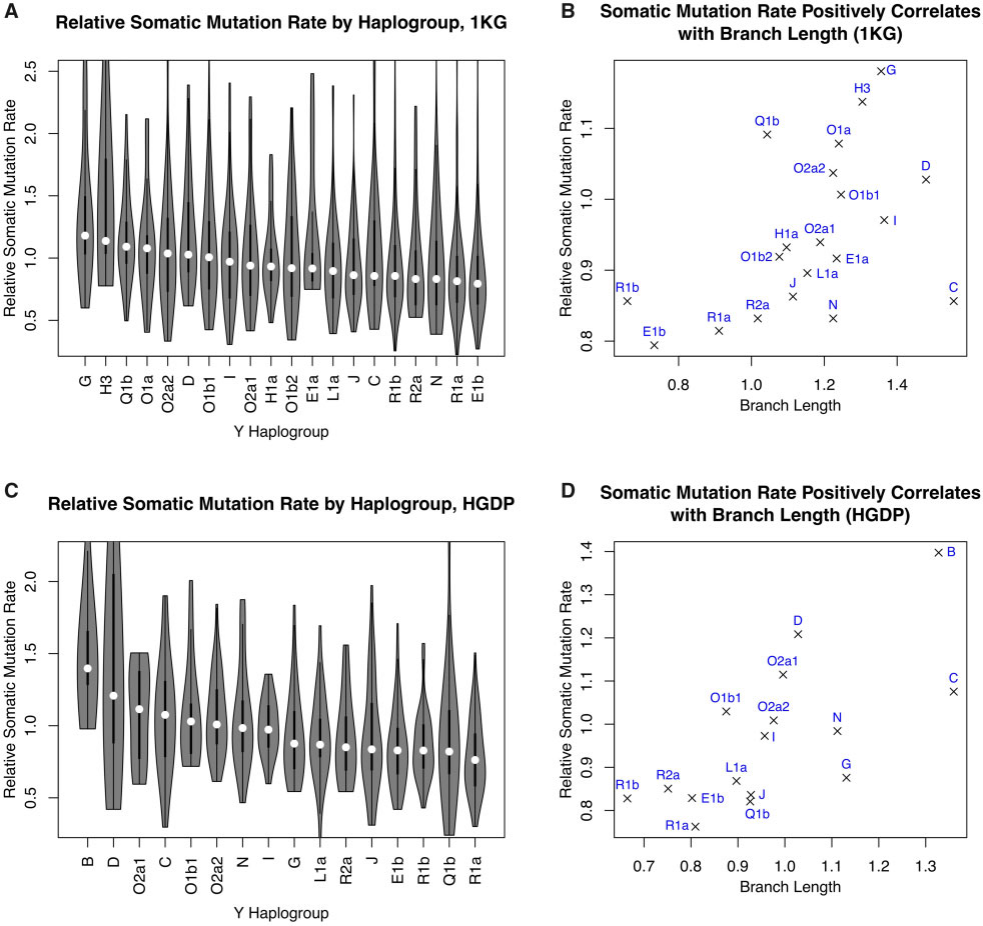
Substantial Y-chromosome somatic mutation rate variation across haplogroups. (*A* and *C*) Distribution of the Y-chromosome somatic (within-cell-line) mutation rate among haplogroups in the 1KG (*A*) and HGDP (*C*) data sets. In both data sets, the mutation rate was significantly variable across haplogroups (see also, supplementary fig. S10, Supplementary Material online). (*B* and *D*) Variation in somatic mutation rate is correlated with branch length heterogeneity in the 1KG (*B*) and HGDP (*D*) data sets, suggesting that interhaplogroup mutation rate variation is a parsimonious explanation for branch length heterogeneity.

We observed significant variation in the Y-chromosome somatic mutation rate among haplogroups using the 1KG data set (fig. 3*A*, *P *= 5.30 × 10^−10^, Kruskal–Wallis rank-sum test). Strikingly, the interhaplogroup variation in somatic mutation rate and phylogenetic branch length were positively correlated (Spearman *ρ* = 0.54, *P *= 1.54 × 10^−2^, fig. 3*B*), suggesting that similar variation in mutation rate is likely also present in the germline. For example, haplogroups E and R had the shortest phylogenetic branch lengths and the lowest somatic mutation rates. Consistent with the findings in the 1KG data set, significant interhaplogroup variation in the somatic mutation rate (*P *= 3.52 × 10^−9^, fig. 3*C*) was observed in the HGDP data set, which was positively correlated with branch length heterogeneity (*ρ* = 0.78, *P *= 2.62 × 10^−3^, fig. 3*D*). These findings were robust to analytical approaches (supplementary note 3, Supplementary Material online).

The extent of interhaplogroup variation in somatic mutation rate was substantial. The haplogroups with the highest and lowest median somatic mutation rate have an 83.3% difference in the HGDP data set (48.6% in the 1KG data set). There was no clear pattern that the mutation rates cluster by phylogeny, suggesting multiple events, at different time scales, affected Y-chromosome mutation rate. For example, assuming that higher mutation rate is ancestral, there were likely multiple slowdown events which occurred independently in the ancestors of haplogroups E and R. Our conclusions were unlikely driven by batch effects (supplementary note 4, Supplementary Material online). In summary, our findings indicate that there is substantial interhaplogroup variation in Y-chromosome mutation rate, and that such variation is a parsimonious explanation for phylogenetic branch length heterogeneity.

This all begs the question—why do Y haplogroups have different rates of mutation? What is the mechanism driving this variation? One plausible mechanism is variation in replication timing across haplogroups. We previously reported interindividual variation in replication timing, and showed that it was associated with genetic variants (rtQTLs) (Koren et al. 2014; Ding et al. 2020). Since replication timing is negatively linked to mutation rate, haplogroups associated with earlier replication may have a lower mutation rate. Y chromosomes have highly variable amounts of heterochromatin (Repping et al. 2006), which is usually late-replicating. One hypothesis would be that Y chromosomes with the greatest abundance of heterochromatin are also the latest replicating. But heterochromatin abundance changes rapidly, and it is not well correlated with haplogroup (Repping et al. 2006), so there are probably other factors, as yet unknown, that account for the among-haplogroup heterogeneity in replication timing and/or mutation rate.

In this study, we generated the first human Y-chromosome replication timing profile (supplementary file 1, Supplementary Material online), which provides quantification of regional mutation rate variation. We also revealed that the Y-chromosome mutation rate varies across haplogroups. This has important implications for evolutionary genetics, since inferences of divergence times could possibly be distorted by variation in mutation rate across haplogroups (supplementary note 5, Supplementary Material online).

## Supplementary Material

Supplementary data are available at *Molecular Biology and Evolution* online.

## Supplementary Material

msaa268_Supplementary_Data
