# Supplementary material for: Mutation Rate Variability across Human Y-Chromosome Haplogroups
Source: Mol Biol Evol. 2020 Oct 13;38(3):1000–5. doi: 10.1093/molbev/msaa268 (PMC7947773; doi:10.1093/molbev/msaa268)
Supplement: msaa268_Supplementary_Data [file msaa268_supplementary_data.zip › Y_suppl_round2_v2.pdf]

Supplementary Material

for

Mutation Rate Variability across Human Y-Chromosome Haplogroups

Authors: Qiliang Ding[1], Ya Hu[1,2], Amnon Koren[1*], Andrew G. Clark[1,3*]

[1] Department of Molecular Biology and Genetics, Cornell University, Ithaca NY 14853

[2] New York Genome Center, New York NY 10013

[3] Department of Computational Biology, Cornell University, Ithaca NY 14853

* Correspondence to: Andrew G. Clark (ac347@cornell.edu) and Amnon Koren (koren@cornell.edu).

Table of Contents

Material and Methods

Supplementary Note 1–5

Supplementary Figure 1–10

Supplementary Table 1 and 2

Supplementary File 1

Materials and Methods

Data sources

We used two whole-genome sequencing datasets: the high-coverage 1000 Genomes Project Phase 3 dataset (hereafter referred to as the 1KG dataset), and the Human Genome Diversity Project dataset (hereafter referred to as the HGDP dataset) (Bergström, et al. 2019). The 1KG and HGDP datasets contain 1,233 and 554 males, respectively. We removed 38 males in the 1KG dataset for suspected Y-chromosome aneuploidy or copy-number alterations. CRAM files for the 1KG and HGDP datasets were obtained from the European Nucleotide Archive (ENA) under accession numbers PRJEB31736 and PRJEB6463, respectively. Y-chromosome VCF file of the 1KG dataset was obtained from the 1000 Genomes FTP site (ftp.1000genomes.ebi.ac.uk/). All genomic coordinates in this study were in hg38.

Y-chromosome haplogroup classification

A list of haplogroup-informative single-nucleotide polymorphisms (SNPs) was obtained from the International Society of Genetic Genealogy (ISOGG) website on July 20, 2019. For each sample, we generated genotypes at the ISOGG SNPs based on CRAM file(s) of the sample. Based on the distribution of haplogroups in our datasets, we assigned samples to one of the following Y haplogroups: B, C, D, E1a, E1b, G, H1a, H3, I, J, L1a, N, O1a, O1b1, O1b2, O2a1, O2a2, Q1b, R1a, R1b, or R2a. Note that we excluded any haplogroup with fewer than 10 samples in our datasets. In addition, because there were fewer than ten samples in the given haplogroup in the given dataset, haplogroup B and haplogroups E1a, H1a, H3, O1a, and O1b2 were excluded from analysis based on the 1KG and HGDP datasets, respectively.

Branch length heterogeneity

In the 1KG dataset, genetic variants were obtained from the public Y-chromosome VCF file. SNPs with the “PASS” flag in the VCF file were used. We excluded all heterozygous genotype calls. A phylogenetic tree was reconstructed using the maximum-likelihood

method in MEGA (Kumar, et al. 2018), with default parameters. The tree was visualized using FigTree v1.4.4 (http://tree.bio.ed.ac.uk/software/figtree/). We used the likelihood-ratio test in MEGA to evaluate evolutionary rate non-uniformity in the reconstructed tree. This test compares likelihood value of the given tree topology with or without constraints of the molecular clock (Kumar, et al. 2018; Stecher, et al. 2020). A haplogroup A0a sample (HG02982) was used as the outgroup. We repeated the test using subsets of samples and sites and found that evolutionary rate uniformity was overwhelmingly rejected (Supplementary Note 1). The RelTime method (Tamura, et al. 2012) was used to estimate branch-specific evolutionary rates on terminal branches, and this was used as a surrogate for branch length. Default parameters were used. For each sample, we computed normalized evolutionary rate by dividing its evolutionary rate by the average of all samples in the given dataset.

In the HGDP dataset, reads mapped to the Y chromosome were extracted from whole-genome sequencing CRAM files, and GATK HaplotypeCaller was used to identify genetic variants (McKenna, et al. 2010). We were unable to run HaplotypeCaller on the highly repetitive region of the Y chromosome beyond 50 Mb. We filtered the call set using low-quality variants (that did not have the “PASS” flag) in the 1KG Y-chromosome VCF file. As was done for the 1KG data set, a maximum-likelihood phylogeny was reconstructed in MEGA and visualized in FigTree v1.4.4. The same likelihood-ratio test as describe above was applied to evaluate evolutionary rate non-uniformity. An individual from haplogroup A1b1 (HGDP01029) was used as the outgroup. Branch-specific evolutionary rates were estimated using the RelTime method. We also computed normalized evolutionary rates as described above.

Of note, genetic variants used in the above analyses must be supported by all reads or have no more than two reads supporting an alternative allele. The median read depth on the Y chromosome was ~15. We compared these genetic variants against the within-cell-line mutations identified (see below for details), and no overlap was found. Thus, the findings on branch length (this section) and on the relative somatic mutation rate (see below for details) were based on different and non-overlapping sets of genetic variants.

Within-cell-line mutations

To identify mutations that appear to be variable within each cell line, the `mpileup` command in samtools (Li, et al. 2009) was used to extract reads from each cell line aligned to the sex chromosomes from CRAM files, requiring both the base quality and mapping quality to be at least 20. A site had to satisfy all following criteria to be considered as a "within-cell-line" mutation: (1) the alternative allele was supported by at least three reads, (2) no more than one read carried any allele other than the reference and alternative alleles, (3) uniquely alignable according to an alignability mask from GenomeSTRiP (Handsaker, et al. 2011), (4) read depth of this site was between the 10th and 90th percentile of all sites on this chromosome in this sample, and (5) the mutation should be absent from other samples or found in only one other sample in the 1KG or HGDP dataset. In total, 96.9% (34,743/35,871) of the within-cell-line mutations were private to one sample. This method was applied to the non-pseudoautosomal regions of the Y chromosome and the 70 to 140 Mb regions of the X chromosome.

To validate within-cell-line mutations, we obtained five samples from the Coriell Institute for Medical Research, namely NA18486, NA18498, NA18917, NA18923, and NA19175, and performed whole-genome resequencing. Reads were aligned to hg38 using BWA (Li and Durbin 2009). We selected 14 within-cell-line mutations for validation, because the alternative alleles at these sites were supported by five or more reads in the 1KG dataset. A mutation was considered as validated if the alternative allele was supported by at least one read (both the base quality and the mapping quality above 20) in the resequencing data.

To evaluate whether alternative alleles at the within-cell-line mutation sites have lower genotype quality (Q) than their respective reference alleles (Anderson-Trocmé, et al. 2019), we randomly selected 10,000 within-cell-line mutation sites, and obtained their genotype quality, stratified by genotype, from CRAM files. To identify Q-associated sites, genotype quality of the reference and alternative alleles were compared using the Wilcoxon rank-sum test for each site. Bonferroni correction was used to correct for multiple testing.

In the lower panels of Figure 2A, we plotted mutation density tracks for within-cell-line and germline mutations. For both types of mutations, the 1KG dataset was used. We counted the number of mutations (within-cell-line or germline) that fall in each replication timing bin. We further plotted the smoothed mutation density series (using penalized smoothing spline, with parameter 10^{-16}).

Y-chromosome replication timing and relationship with the mutation spectrum

As previously described (Koren, et al. 2014), we inferred Y-chromosome replication timing in lymphoblastoid cell lines (LCLs) using the 1KG dataset. Specifically, for each sample, we ran GenomeSTRiP to extract Y-chromosome read depths in 10 kb windows of uniquely alignable sequence (Handsaker, et al. 2011). We then removed outlier data points. The data was further smoothed using a smoothing spline with parameter 10^{-16} and normalized to *Z*-score (mean of 0 and standard deviation of 1). The smoothed and normalized read depth data was used as the replication timing profile for further analyses. We averaged replication timing profiles for all samples in the 1KG dataset to generate a high-quality consensus Y-chromosome replication timing profile in LCLs.

We explored the relationship between replication timing and the distribution of within-cell-line mutations. For each replication timing window, we counted the number of within-cell-line mutations found within the given window in 1KG samples. Replication timing windows were non-overlapping. We also implemented a binning-based approach (Stamatoyannopoulos, et al. 2009; Koren, et al. 2012; Woo and Li 2012) to study the relationship between mutation and replication timing. Specifically, replication timing windows were assigned to one of 25 equal-sized bins based on their timing. We then calculated and plotted mean replication timing and mutation counts for windows in each bin. This analysis was also performed using within-cell-line mutation data of the HGDP dataset. We repeated these analyses using germline mutation data (polymorphic sites in the 1KG dataset, obtained from the publicly-available Y-chromosome VCF file). For each analysis, we observed the qualitative relationship between mutation and replication timing and noted that there was universally a negative trend.

We also studied the mutation spectrum of germline and within-cell-line mutations on the Y chromosome. As previously described, we categorized mutations based on the type of substitution and the 1-bp up/downstream nucleotide context (Alexandrov, et al. 2020). The mutation counts were normalized by the number of possible mutations under each category on chromosome Y (also known as the "opportunity matrix") and plotted using R package `signeR` (Rosales, et al. 2016) and subsequently edited. Due to the relative scarcity of mutations, we only plotted mutational spectra and did not perform mutational signature discovery.

Relative Y-chromosome mutation rate

For each sample, we computed the relative Y-chromosome mutation rate as the ratio of within-cell-line mutation counts (calculated as genetic distance using the Kimura two-parameter model) of the Y chromosome versus the X chromosome. For each sample, the number of within-cell-line transitions and transversions were summed up and used to calculate the relative Y-chromosome mutation rate. We excluded 13 samples in the 1KG dataset and three samples in the HGDP dataset for this analysis, since they had unusually high numbers of within-cell-line mutations on the X and/or Y chromosome.

To show that the finding of inter-haplogroup variation in the relative somatic mutation rate was not driven by normalization in calculating the rate, we assigned samples to one of five groups based on the within-cell-line mutation counts on the X chromosome. We then directly compared within-cell-line mutation counts on the Y chromosome between haplogroup E or R samples and non-haplogroup E/R samples within each group. Using this approach, we minimized the necessity of normalization (similar mutation counts on the X). Haplogroups E and R were selected due to their lower somatic relative mutation rate (Figure 3). Statistical significance was assessed using the Wilcoxon rank-sum test.

Mutation rate adjustment schedule

Two Y-chromosome mutation rate estimates (Xue, et al. 2009; Helgason, et al. 2015) are commonly used in current sequencing-based studies. The Xue et al. rate was

estimated with a haplogroup O2a1 pedigree. The Helgason et al. study used 274 pedigrees, among which 96, 95, 66, 16, and 1 belong to haplogroup R1b, I, R1a, Q, and E1b, respectively. To identify haplogroups with significantly different mutation rate than the Xue et al. rate, we used the Wilcoxon rank-sum test to assess significance of difference in the relative somatic mutation rates between the given haplogroup and haplogroup O2a1. Nominal $p < 0.05$ was considered significant. We used the same method to identify haplogroups with significantly different mutation rate than the Helgason et al. rate, replacing haplogroup O2a1 with a group of 130 samples (45, 45, 31, 8, and 1 belong to haplogroup R1b, I, R1a, Q, and E1b, respectively). Sample size of this group was smaller than the Helgason et al. study due to limited availability of haplogroup I samples in our dataset, but the haplogroup composition was identical to that of the Helgason et al. study. Both the 1KG and HGDP datasets were used in the computations.

Supplementary Notes

Supplementary Note 1: Y-chromosome Evolutionary Rate Non-uniformity was Robust to Subsampling of Individuals and Sites

In this study, the hypothesis of evolutionary rate uniformity was rejected for Y-chromosome sequences from the 1KG and HGDP datasets. Nevertheless, because there were a large number of individuals and nucleotide sites in these datasets, and rate uniformity may not hold exactly, it is imperative to evaluate whether these conclusions were robust to subsampling of individuals and sites.

Specifically, we first selected n samples per haplogroup, then randomly selected m% of sites, and performed the test of rate uniformity on the subsampled data. We repeated the process 100 times for each (n, m) combination, and counted the proportion of nominally significant ($p < 0.05$) tests. As shown in Table S1, in the 1KG dataset, even when we only use five samples per haplogroup (total: 100 samples, 8.8% of the full dataset) and 5% of the sites, 86% of the tests have $p < 0.05$, which is much greater than expected under chance. Observations in Table S1 indicate that evolutionary rate uniformity was overwhelmingly rejected even when a small fraction of samples and sites were used. These findings were reproduced using the HGDP dataset (Table S2). Taken together, these observations support our claim of evolutionary rate heterogeneity in the human Y-chromosome phylogeny.

Supplementary Note 2: Relationship between Mutations and Replication Timing

In the main text, using a binning-based approach, we observed a negative relationship between within-cell-line mutations and replication timing in the 1KG dataset (i.e. later replicating regions display greater numbers of within-cell-line mutations, Figure 2B). Here we demonstrate that this finding is robust to datasets and analytic approaches.

We first performed a window-based analyses. For each replication timing window, we counted within-cell-line mutations located in the given window. We then plotted mutation counts against replication timing. We uncovered a negative trend between within-cell-line mutation count and replication timing in the 1KG dataset (Figure S1A). This finding was reproduced in the HGDP dataset (Figure S1B) and using germline mutation data (Figure S1C). We reproduced the negative trend between replication timing and within-cell-line mutation using the binning-based approach in the 1KG (Figure 2) and HGDP (Figure S2) datasets.

We found that the mutation spectra of within-cell-line mutations (Figure S3A) and germline mutations (Figure S3B) were largely similar. The most common mutation types were C>T mutations at NCG sites, likely representing deamination of 5-methylcytosine to thymine at CpG islands. Notably, these mutations were more common in the germline. Because cell lines were usually frozen unless during culturing, it is expected that fewer mutations would arise from spontaneous processes (such deamination of methylated cytosine) in cell lines. The two mutational spectra were otherwise very similar visually, indicating similar mutational processes. Notably, we did not observe visible contributions from known sequencing artifacts (Alexandrov, et al. 2020). Genotype quality score did not substantially differ between reference and alternative alleles at within-cell-line mutation sites (median quality scores for both alleles were 30, Figure S4), with only 0.16% of the sites show association with genotype quality (Anderson-Trocmé, et al. 2019). These findings support that the within-cell-line mutations were true genetic variants.

The within-cell-line mutations could come from two sources: (1) *in vivo* along the hematopoietic linage and (2) *in vitro* during cell line propagation. In this study, we were unable to distinguish the two sources and infer what proportion of the within-cell-line

mutations were *in vitro* and *in vivo*. Nonetheless, lacking this information does not affect our conclusions, because mutations occurred both *in vivo* and *in vitro* are informative for our purposes. Our hypothesis is that Y chromosomes from some haplogroups were more (or less) mutagenic than others. Because major mutational mechanisms (e.g., replicative error) do not fundamentally differ in hematopoietic cells and LCLs, we expect Y chromosomes from the more (or less) mutagenic haplogroups continue to produce more (or fewer) mutations *in vitro*. Furthermore, both types of mutations were not affected by non-genetic confounders and are therefore suitable for our analyses. In addition, empirically, within-cell-line mutations (union of *in vitro* and *in vivo* mutations) exhibit negative relationship with LCL replication timing (Figure 2B), suggesting that these mutations were true genetic variants.

Supplementary Note 3: Findings based on Relative Somatic Mutation Rate were Robust to Analytical Approaches

Figure S5 shows the distribution of raw within-cell-line mutation counts (on the X and Y chromosomes) used to estimate the relative somatic mutation rate (i.e., Y/X ratio). In the 1KG dataset, the median number of within-cell-line mutations on the Y chromosome was 15 (interquartile range: 11–21), while the median number of such mutations on the X chromosome (70–140 Mb region) was 50 (interquartile range: 33–70.75). In the HGDP data, the median number of such mutations on the Y was 24 (interquartile range: 18–32), while the median number of such mutations on the X (70–140 Mb region) was 79 (interquartile range: 58–106).

One may question whether the computation of Y/X ratio artificially created inter-haplogroup variation in the relative somatic mutation rate. To address this issue, instead of calculating a Y/X ratio, we assigned samples into one of five equal-sized groups based on within-cell-line mutation count on the X (i.e., samples within each group had similar X mutation count). Within each group, we compared within-cell-line mutation counts on the Y across haplogroups, without adjustment using within-cell-line mutation counts on the X. Using this approach, we avoided the issue of comparing Y/X ratios. Due to the reduced power (less samples per group), we focused on reproducing the lower mutation rate in haplogroups E and R compared with other haplogroups (Figure 3). In the 1KG dataset, this pattern was reproduced in four out of the five groups (Figure S6). Similarly, this pattern was also reproduced in four out of the five groups in the HGDP dataset (Figure S7). These results suggest that the observed difference in the relative somatic mutation rate across haplogroups was not artifact due to comparison on Y/X ratios.

Further, the correlation between branch length (branch-specific evolutionary rate estimated by RelTime) and the relative somatic mutation rate holds when using individual measurements (instead of summarized by haplogroup, Figure 3) in correlation analyses (1KG: Spearman $\rho = 0.22$, $p = 1.70\times10^{-14}$, HGDP: $\rho = 0.13$, $p = 3.73\times10^{-3}$, Figure S8).

Supplementary Note 4: Our Conclusions were Not Likely Driven by Batch Effects

It is essential to ensure that our results were not driven by batch effects. The data and analyses were not likely affected by batch effects for several reasons. First, data processing and analyses in our study were blinded to haplogroup. Second, cell lines in both datasets (1KG and HGDP) were maintained and propagated at a central laboratory (1KG: the Coriell Institute for Medical Research; HGDP: Centre d'Étude du Polymorphisme Humain in France), and there was no report that the laboratory subjected the cell lines to different treatments by Y-chromosome haplogroup. Third, sequencing data for both datasets were generated by a single center (1KG: the New York Genome Center, HGDP: the Wellcome Sanger Institute) in a single study with uniform protocol and sequencing platform. More importantly, our findings were replicated in two independent datasets (from different groups at different sequencing centers with cell lines collected and maintained by different sources). Because it is unlikely that the two independent datasets shared similar technical confounders, our findings were likely driven by biological reasons.

In addition, even if LCLs from different haplogroups were propagated for different numbers of passages (leading to different numbers of accumulated within-cell-line mutations), this issue would be effectively mitigated by our study design. First, the findings on branch length heterogeneity would not be affected because they did not rely on within-cell-line mutations. Second, we normalized within-cell-line mutation counts on the Y chromosome using within-cell-line mutation counts on the X chromosome (the relative somatic mutation rate) to correct for technical confounders. Technical issues, e.g., time spent in cell line propagation, would affect mutation accumulations on all chromosomes (including the X and Y), instead of affecting mutation accumulation on the Y chromosome only. This was supported by the observation that the number of mutations on the X and Y were largely correlated (1KG: $\rho = 0.72$; HGDP: $\rho = 0.64$, Figure S5 above).

To further show that the variation in the relative somatic mutation rate among haplogroups was robust to the number of cell culturing passages, we stratified the 1KG samples into five equal-sized groups based on the within-cell-line mutation counts on

the X. Samples within each group (i.e., similar mutation counts on the X) would have comparable exposure to factors affecting mutation accumulation (such as time spent during cell line propagation) regardless of Y-chromosome haplogroup. Within each group, we compared the count of within-cell-line mutations on the Y chromosome between haplogroup E or R samples and non-haplogroup E/R samples. We selected these two groups because of the observed lower relative somatic mutation rate for haplogroup E and R samples (Figure 3). Statistical power was limited in this analysis because of grouping (fewer samples). Nevertheless, we still reproduced the pattern of significantly lower Y-chromosome within-cell-line mutation counts for haplogroup E or R samples in four of the five groups (Figure S6). Similar findings were observed in the HGDP dataset, in which we were also able to reproduce the pattern in four of the five groups (Figure S7). Therefore, we demonstrated that the inter-haplogroup variation in the relative somatic mutation rate holds, even after restricting analyses to samples from different haplogroups that had similar exposure to factors affecting mutation accumulation.

In the 1KG dataset, samples were collected in populations (for example, YRI – Yoruba in Ibadan, Nigeria) by local research institutions. It is possible that minute differences in sample collection and handling may introduce batch effect. To explore this issue, we stratified samples by population, and compared the relative somatic mutation rate between haplogroup E or R samples and non-haplogroup E/R samples. We limited this analysis to populations with at least 30% but no more than 70% of haplogroup E and R samples. In all eight populations examined, the median relative somatic mutation rate of haplogroup E or R samples was lower than that of non-haplogroup E/R samples (Figure S9). This supports that our conclusion on inter-haplogroup variation in Y-chromosome mutation rate holds even when stratified by population. We did not assess significance in this analysis due to the extremely limited statistical power (very small sample size – median: 49 samples, i.e., < 5% of total samples).

Taken together, we showed that, both theoretically and empirically, our conclusions were not driven by batch effects.

Supplementary Note 5: Suggestions when Applying Two Commonly Used Y-chromosome Mutation Rates to Certain Haplogroups

Two Y-chromosome mutation rate estimates were commonly used in current studies (Xue, et al. 2009; Helgason, et al. 2015). The Xue et al. rate was based on a haplogroup O2a1 pedigree. The Helgason et al. rate was based on 274 pedigrees (from haplogroups E1b, I, Q, R1a and R1b). In this study, we found inter-haplogroup variation in Y-chromosome mutation rate. Thus, it may no longer be suitable to apply the same mutation rate across various haplogroups.

For the two mutation rates mentioned above, we identified haplogroups with significantly different relative somatic mutation rate than the haplogroup(s) from which the mutation rate was estimated (see below). For example, haplogroup O2a1 had significantly higher somatic mutation rate than haplogroup R1a. If another study applies the Xue et al. (2009) rate to haplogroup R1a samples, we recommend adding a sentence stating that the actual mutation rate in haplogroup R1a may be lower than the Xue et al. (2009) rate, and therefore divergence times may be underestimated. It would be ideal to provide a quantitative adjustment factor for various haplogroups. However, we elected to take a qualitative approach, because our point estimates on adjustment factors may not be precise, and thus may introduce additional bias in divergence time estimations. Further studies with larger sample sizes and/or based on pedigrees, with careful control to non-genetic confounders (e.g., paternal age) would be required to provide quantitative adjustment factors.

For the Xue et al. (2009) rate, we identified the following haplogroups having significantly lower relative somatic mutation rate: E1b, R1a, and R1b. For these haplogroups, their actual mutation rate may be lower than the Xue et al. (2009) rate, and thus divergence times may be underestimated. For the Xue et al. (2009) rate, haplogroup B has significantly higher relative somatic mutation rate. For haplogroup B, its actual mutation rate may be higher than the Xue et al. (2009) rate, and thus divergence times may be overestimated.

For the Helgason et al. (2015) rate, we identified the following haplogroups having significantly lower relative somatic mutation rate: E1b and R1a. For these haplogroups,

their actual mutation rate may be lower than the Helgason et al. (2015) rate, and thus divergence times may be underestimated. For the Helgason et al. (2015) rate, haplogroup B has significantly higher relative somatic mutation rate. For haplogroup B, its actual mutation rate may be higher than the Helgason et al. (2015), and therefore divergence times may be overestimated.

Supplementary Figures

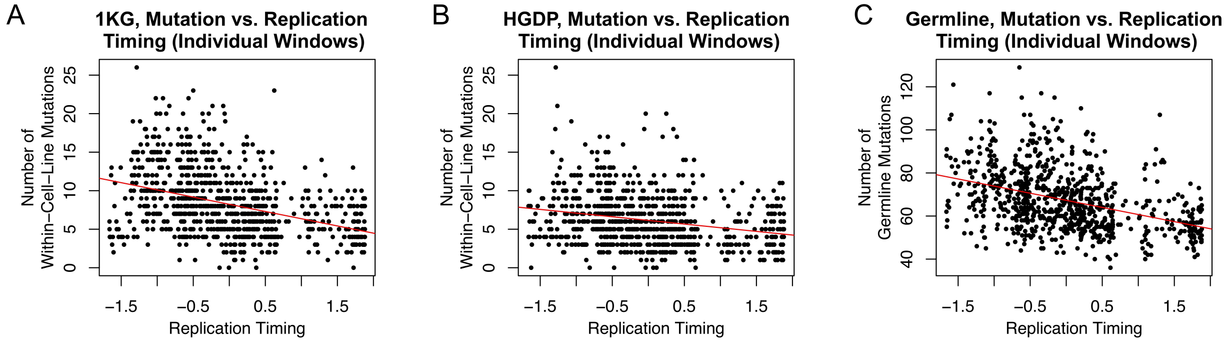

Figure S1. Relationship between Mutations and Replication Timing.

(A, B) Within-cell-line mutations in the 1KG (A) and HGDP datasets. (C) Germline mutations, using SNPs detected in the 1KG dataset as a proxy. Each data point represents one replication timing window. We counted mutations located within each replication timing windows. Red: linear trend line. Negative trend was observed in all three plots.

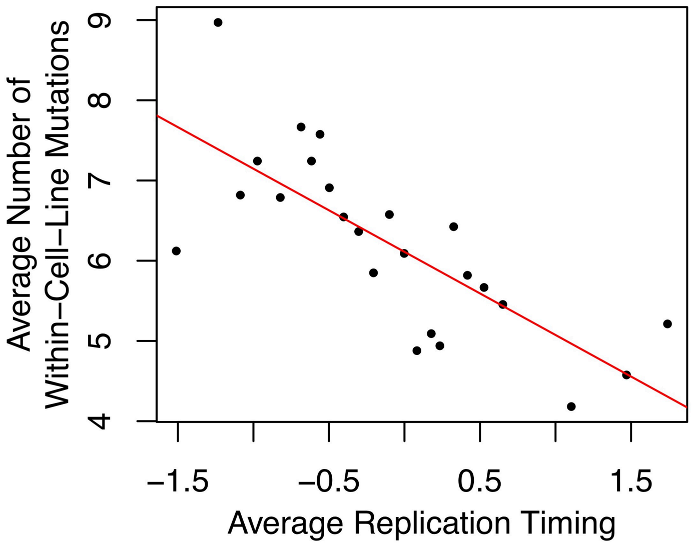

Figure S2. Relationship Between Replication Timing and Within-cell-line Mutations in the HGDP Dataset, in 25 Bins.

See Figure 2 for more details. Each data point represents one bin. Red: linear trend line.

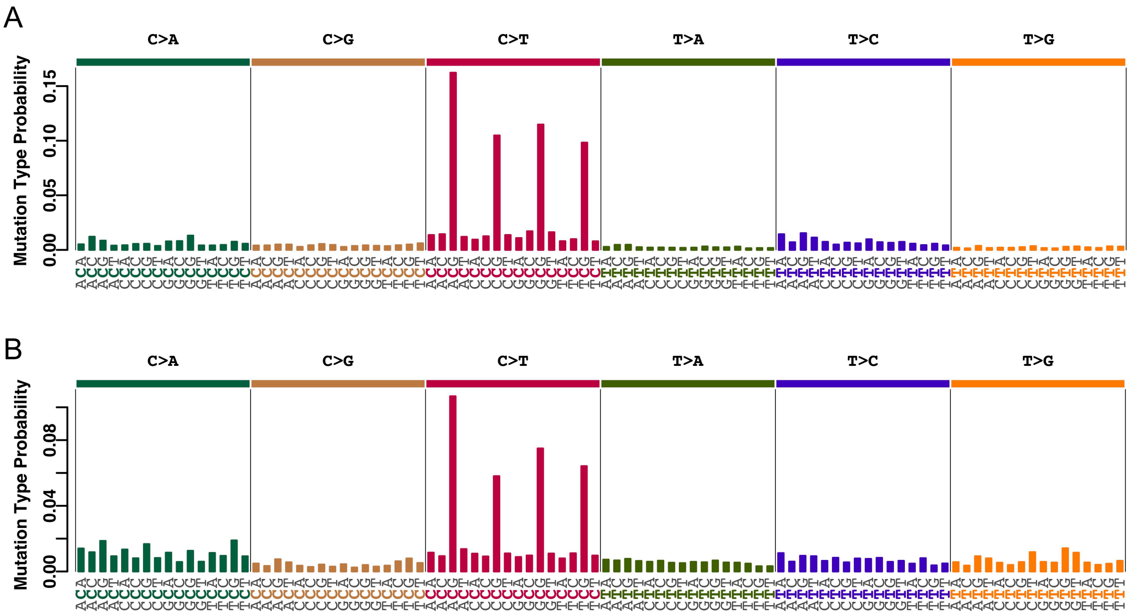

Figure S3. Mutation Spectrum of Germline (A) and Within-cell-line Mutations (B).

The most common mutation types were C>T mutations at NCG sites, likely from deamination of 5-mC at CpG islands. Otherwise the two mutation spectra were very similar. No visible contribution from known sequencing artifacts was observed.

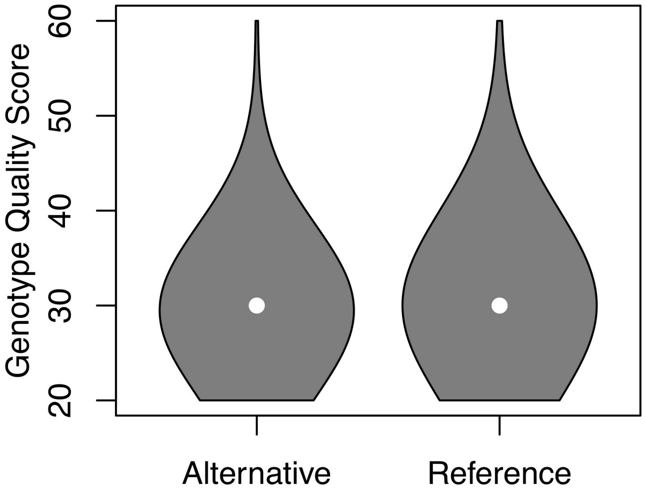

Figure S4. Genotype Quality Scores were Similar between Reference and Alternative Alleles at Within-cell-line Mutation Sites.

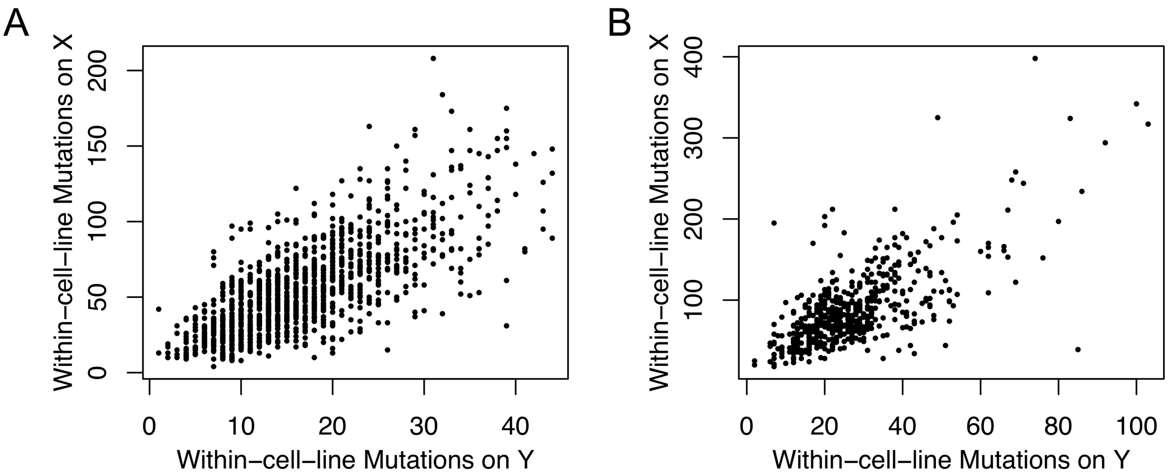

Figure S5. Distribution of Within-cell-line Mutation Counts on the X and Y Chromosomes across 1KG (A) and HGDP (B) Samples.

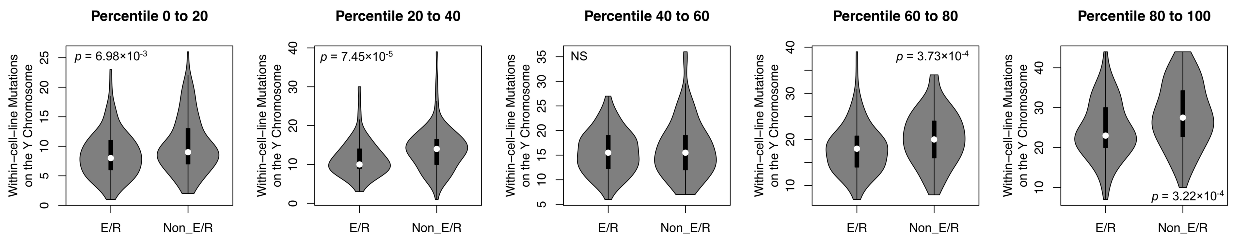

Figure S6. Comparison of Within-cell-line Mutation Counts on the Y Chromosome between Haplogroups using the 1KG Dataset, Stratified by Within-cell-line Mutation Counts on the X Chromosome.

Samples in the 1KG dataset were assigned to one of five groups based on their within-cell-line mutation counts on the X, shown as the five panels in the figure. Within each group, we compared within-cell-line mutation counts on the Y chromosome between samples in haplogroup E or R ("E/R" in the plot) and non-haplogroup E/R samples ("Non_E/R" in the plot). Statistical significance was assessed by one-tailed Wilcoxon rank-sum test, and *p*-values were shown in the plots. NS: not significant. In four out of the five groups, samples in haplogroup E or R have significantly lower within-cell-line mutation counts on the Y chromosome, consistent with the findings in Figures 3A and 3C (based on Y/X ratios, i.e., relative somatic mutation rate).

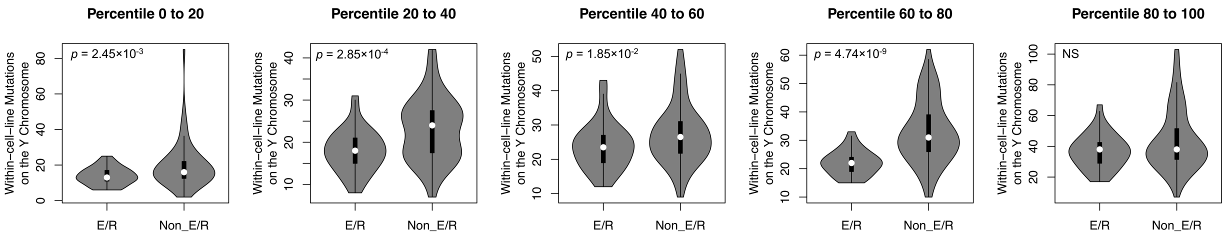

Figure S7. Comparison of Within-cell-line Mutation Counts on the Y Chromosome between Haplogroups using the HGDP Dataset, Stratified by Within-cell-line Mutation Counts on the X Chromosome.

See Figure S6 for detailed explanations.

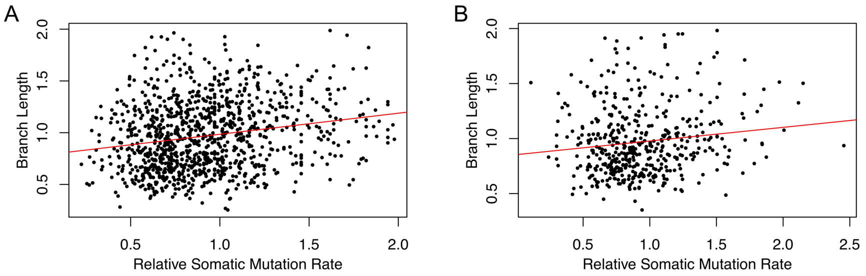

Figure S8. Correlation between Branch Length and Relative Somatic Mutation Rate in the 1KG (A) and HGDP (B) Datasets, using Individual Measurements.

Red line: fitted linear relationship. In both plots, there were significant positive correlation between branch length and relative somatic mutation rate.

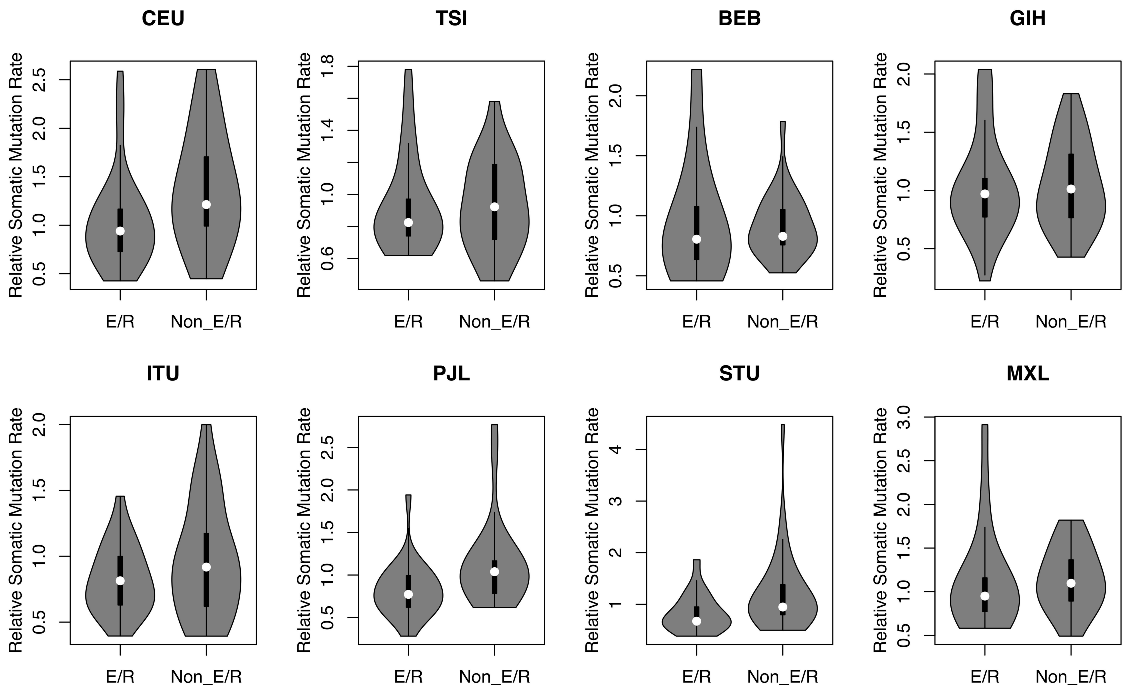

Figure S9. Comparison of the Y-chromosome Relative Somatic Mutation Rate between Haplogroups in the 1KG Dataset, Stratified by Population.

Within each population, we compared Y-chromosome relative somatic mutation rate between samples in haplogroup E or R (“E/R”) and non-haplogroup E/R samples (“Non_E/R”). This analysis was limited to populations with > 30% but < 70% of haplogroup E and R samples. In all eight populations, samples in haplogroup E or R have lower median Y-chromosome relative somatic mutation rate, consistent with the findings in Figure 3.

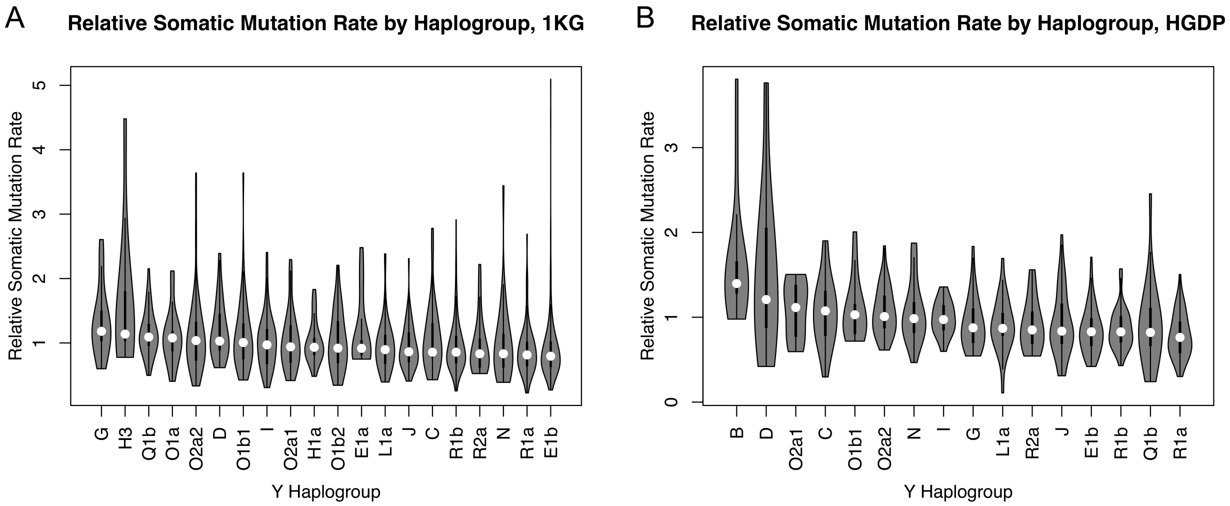

Figure S10. Distribution of the Y-chromosome Somatic Mutation Rate among Haplogroups in the 1KG (A) and HGDP (B) Datasets, Full Plots.

Also see Figures 3A and 3C for details.

Supplementary Tables

Table S1. Test of Evolutionary Rate Uniformity Using Subsets of Samples and Sites in the 1KG Dataset.

We randomly selected *n* samples per haplogroup (rows) and *m%* of sites (columns) and performed the test of rate uniformity. The process was repeated 100 times for each (*n, m*) combination. The tests were performed in MEGA using likelihood-ratio test (comparing the likelihood of the known tree topology with and without constraints of molecular clock). A haplogroup A sample was used as the outgroup. Shown in the table are the proportion of tests with nominal $p < 0.05$. The hypothesis of evolutionary rate uniformity was overwhelmingly rejected even when a small fraction of samples and sites were used.

		Proportion of Sites Selected			
		5%	10%	20%	50%
Number of Samples Selected per Haplogroup (Total Number of Samples)	1 (20)	45%	80%	97%	100%
	3 (60)	67%	82%	98%	100%
	5 (100)	86%	92%	99%	100%
	10 (200)	97%	100%	100%	100%
	20 (384)	100%	100%	100%	100%

Table S2. Test of Evolutionary Rate Uniformity Using Subsets of Samples and Sites in the HGDP Dataset.

See Table S1 for notes and explanations.

		Proportion of Sites Selected			
		5%	10%	20%	50%
Number of Samples Selected per Haplogroup (Total Number of Samples)	1 (16)	89%	100%	100%	100%
	3 (48)	98%	100%	100%	100%
	5 (80)	100%	100%	100%	100%
	10 (160)	100%	100%	100%	100%
	20 (281)	100%	100%	100%	100%

Supplementary Files

Supplementary File 1. Consensus Y-chromosome replication timing profile in LCL. The first and second columns are genomic position (in hg38) and replication timing, respectively.

References

Alexandrov LB, Kim J, Haradhvala NJ, Huang MN, Tian Ng AW, Wu Y, Boot A, Covington KR, Gordenin DA, Bergstrom EN, et al. 2020. The repertoire of mutational signatures in human cancer. Nature 578:94-101.

Anderson-Trocmé L, Farouni R, Bourgey M, Kamatani Y, Higasa K, Seo J-S, Kim C, Matsuda F, Gravel S. 2019. Legacy Data Confound Genomics Studies. Molecular Biology and Evolution 37:2-10.

Bergström A, McCarthy SA, Hui R, Almarri MA, Ayub Q, Danecek P, Chen Y, Felkel S, Hallast P, Kamm J, et al. 2019. Insights into human genetic variation and population history from 929 diverse genomes. bioRxiv:674986.

Handsaker RE, Korn JM, Nemesh J, McCarroll SA. 2011. Discovery and genotyping of genome structural polymorphism by sequencing on a population scale. Nature Genetics 43:269-276.

Helgason A, Einarsson AW, Guðmundsdóttir VB, Sigurðsson Á, Gunnarsdóttir ED, Jagadeesan A, Ebenesersdóttir SS, Kong A, Stefánsson K. 2015. The Y-chromosome point mutation rate in humans. Nature Genetics 47:453-457.

Koren A, Handsaker RE, Kamitaki N, Karlic R, Ghosh S, Polak P, Eggan K, McCarroll SA. 2014. Genetic variation in human DNA replication timing. Cell 159:1015-1026.

Koren A, Polak P, Nemesh J, Michaelson JJ, Sebat J, Sunyaev SR, McCarroll SA. 2012. Differential relationship of DNA replication timing to different forms of human mutation and variation. American Journal of Human Genetics 91:1033-1040.

Kumar S, Stecher G, Li M, Knyaz C, Tamura K. 2018. MEGA X: Molecular Evolutionary Genetics Analysis across Computing Platforms. Molecular Biology and Evolution 35:1547-1549.

Li H, Durbin R. 2009. Fast and accurate short read alignment with Burrows-Wheeler transform. Bioinformatics 25:1754-1760.

Li H, Handsaker B, Wysoker A, Fennell T, Ruan J, Homer N, Marth G, Abecasis G, Durbin R, Genome Project Data Processing S. 2009. The Sequence Alignment/Map format and SAMtools. Bioinformatics 25:2078-2079.

McKenna A, Hanna M, Banks E, Sivachenko A, Cibulskis K, Kernytsky A, Garimella K, Altshuler D, Gabriel S, Daly M, et al. 2010. The Genome Analysis Toolkit: a MapReduce framework for analyzing next-generation DNA sequencing data. Genome Research 20:1297-1303.

Rosales RA, Drummond RD, Valieris R, Dias-Neto E, da Silva IT. 2016. signeR: an empirical Bayesian approach to mutational signature discovery. Bioinformatics 33:8-16.

Stamatoyannopoulos JA, Adzhubei I, Thurman RE, Kryukov GV, Mirkin SM, Sunyaev SR. 2009. Human mutation rate associated with DNA replication timing. Nature Genetics 41:393-395.

Stecher G, Tamura K, Kumar S. 2020. Molecular Evolutionary Genetics Analysis (MEGA) for macOS. Molecular Biology and Evolution 37:1237-1239.

Tamura K, Battistuzzi FU, Billing-Ross P, Murillo O, Filipski A, Kumar S. 2012. Estimating divergence times in large molecular phylogenies. Proceedings of the National Academy of Sciences 109:19333.

Woo YH, Li W-H. 2012. DNA replication timing and selection shape the landscape of nucleotide variation in cancer genomes. Nature Communications 3:1004.

Xue Y, Wang Q, Long Q, Ng BL, Swerdlow H, Burton J, Skuce C, Taylor R, Abdellah Z, Zhao Y, et al. 2009. Human Y chromosome base-substitution mutation rate measured by direct sequencing in a deep-rooting pedigree. Current Biology 19:1453-1457.
